# (*E*)-2-[(2,4-Dihy­droxy­benzyl­idene)aza­nium­yl]-3-(1*H*-indol-3-yl)propano­ate monohydrate

**DOI:** 10.1107/S1600536811028200

**Published:** 2011-07-23

**Authors:** Salah Ahmed Ba-Salamah, Naser Eltaher Eltayeb, Siang Guan Teoh, Kong Mun Lo

**Affiliations:** a School of Chemical Sciences, Universiti Sains Malaysia, Minden, Penang, Malaysia; b Department of Chemistry, International University of Africa, Sudan; c Chemistry Department, Faculty of Science, University of Malaya, Malaysia

## Abstract

In the zwitterionic title compound, C_18_H_16_N_2_O_4_·H_2_O, the dihedral angle between the planes of the benzene and indole rings is 39.20 (8)°. An intra­molecular N—H⋯O hydrogen bond generates an *S*(6) ring motif. In the crystal, inter­molecular hy­droxy and water O—H⋯O(carboxyl­ate) and N^+^—H⋯O(carboxyl­ate) and indole N—H⋯O(water) hydrogen bonds give a three-dimensional structure.

## Related literature

For related structures, see: Grant *et al.* (1999 ▶); Emge *et al.* (2000 ▶). For the anti­cancer activity of Schiff bases, see: Dao *et al.* (2000 ▶), for their anti-HIV activity, see: Sriram *et al.* (2006 ▶) and for their anti­bacterial and anti­fungal activity, see: Karthikeyan *et al.* (2006 ▶). For analytical applications, see: Eltayeb & Ahmed (2005*a* ▶,*b* ▶). For standard bond lengths, see: Allen *et al.* (1987 ▶).
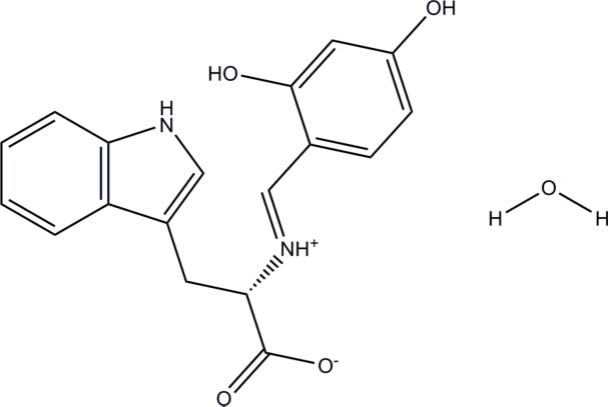


## Experimental

### 

#### Crystal data

C_18_H_16_N_2_O_4_·H_2_O
*M* *_r_* = 342.34
Orthorhombic, 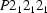

*a* = 8.4214 (3) Å
*b* = 10.6787 (4) Å
*c* = 18.9554 (8) Å
*V* = 1704.65 (11) Å^3^
*Z* = 4
Mo *K*α radiation
μ = 0.10 mm^−1^
*T* = 100 K
0.20 × 0.10 × 0.10 mm

#### Data collection

Bruker SMART APEXII CCD area-detector diffractometer
Absorption correction: multi-scan (*SADABS*; Sheldrick, 1996) ▶ *T*_min_ = 0.618, *T*_max_ = 0.746
15797 measured reflections
3904 independent reflections
3587 reflections with *I* > 2σ(*I*)
*R*_int_ = 0.035

#### Refinement

*R*[*F* ^2^ > 2σ(*F* ^2^)] = 0.034
*wR*(*F* ^2^) = 0.085
*S* = 1.03
3904 reflections
244 parameters
H atoms treated by a mixture of independent and constrained refinement
Δρ_max_ = 0.25 e Å^−3^
Δρ_min_ = −0.16 e Å^−3^

### 

Data collection: *APEX2* (Bruker, 2008 ▶); cell refinement: *SAINT* (Bruker, 2008 ▶); data reduction: *SAINT*; program(s) used to solve structure: *SHELXS97* (Sheldrick, 2008 ▶); program(s) used to refine structure: *SHELXL97* (Sheldrick, 2008 ▶); molecular graphics: *SHELXTL* (Sheldrick, 2008 ▶); software used to prepare material for publication: *publCIF* (Westrip, 2010 ▶).

## Supplementary Material

Crystal structure: contains datablock(s) I, global. DOI: 10.1107/S1600536811028200/zs2121sup1.cif

Structure factors: contains datablock(s) I. DOI: 10.1107/S1600536811028200/zs2121Isup2.hkl

Supplementary material file. DOI: 10.1107/S1600536811028200/zs2121Isup3.cml

Additional supplementary materials:  crystallographic information; 3D view; checkCIF report

## Figures and Tables

**Table 1:** Hydrogen-bond geometry (Å, °)

*D*—H⋯*A*	*D*—H	H⋯*A*	*D*⋯*A*	*D*—H⋯*A*
O4—H4⋯O2^i^	0.84	1.76	2.5966 (15)	178
O3—H3⋯O1^ii^	0.84	1.72	2.5605 (15)	176
O3—H3⋯O2^ii^	0.84	2.64	3.1526 (14)	121
N2—H2*A*⋯O3	0.87 (2)	2.082 (19)	2.6642 (15)	123.9 (16)
O5—H5*B*⋯O2^iii^	0.80 (3)	2.19 (3)	2.9438 (17)	157 (2)
N1—H1*A*⋯O5	0.87 (2)	2.10 (2)	2.9441 (19)	164 (2)

Symmetry codes: (i) 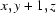; (ii) 
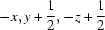
; (iii) 
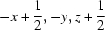
.

**Fig. 1.**
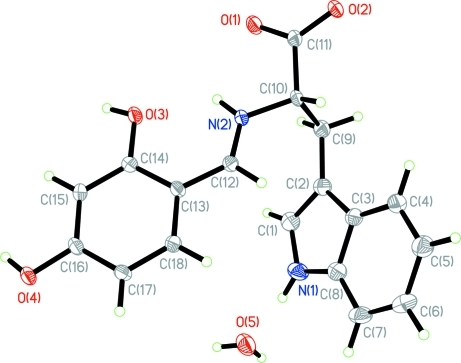
The molecular structure of the title compound, with atom labels and 50% probability ellipsoids for non-H atoms.

**Fig. 2.**
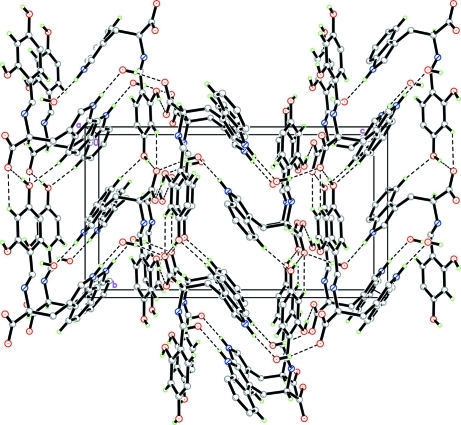
The crystal packing of title compound, viewed down a axis.

## supplementary crystallographic information

### Comment

Schiff bases have received much attention because of their potential applications, with some of these compounds exhibiting various pharmacological activities, as noted by their anticancer (Dao *et al.*, 2000), anti-HIV (Sriram *et al.*, 2006), antibacterial and antifungal (Karthikeyan *et al.*, 2006) properties. In addition, some of them may be used as analytical reagents for the determination of trace elements (Eltayeb & Ahmed, 2005*a*,*b*). In this paper, we report the crystal structure of the title compound C_18_H_16_N_2_O_4_.H_2_O (Fig. 1), obtained by the reaction of tryptophan and 2,4-dihydroxybenzaldehyde.

The asymmetric unit of the title compound (Fig. 1) consists of one zwitterionic *E*)-2-(2,4-dihydroxybenzylideneammonio)-3-(1*H*- indol-3-yl)propanoate molecule and one molecule of water. Bond lengths and angles have normal values (Allen *et al.*, 1987). The dihedral angle between the planes of the benzene and the indole rings in the organic molecule is 39.55 (6)°. Intramolecular N—H···O hydrogen bonds generate S(6) ring motifs. The C13 in the six-membered ring and C2 in the nine-membered ring are connected together by a chain of four atoms, C12/N2/C10/C9 which has torsion angle 78.95 (17) °. The torsion angles of the chain N2/C10/C9/C2 and C10/N2/C12/C13 are -57.03 (16) ° and -174.50 (13) °, respectively. In the crystal structure, the molecules are linked by intermolecular hydroxy O—H···O_carboxylate_ hydrogen bonds into chains which extend along the *b* axis and peripherally by N^+^—H··· O_carboxylate_ hydrogen bonds (Table 1 and Fig. 2). The three-dimensional structure is also stabilized by the indole N—H···O_water_ and water O—H···O_carboxylate_ associations.

The absolute configuration could not be determined definitively [Flack parameter -0.02 (8) (Flack, 1983)] but C10 (*S*) was assumed for the title compound.

### Experimental

To a stirred solution of 2 mmol of tryptophan (0.416 g) in 20 ml of (3:1) methanol-water solvent was added 2 mmol of 2,4-dihydroxybenzaldehyde (0.282 g), giving a light pink clear solution. The mixture was refluxed with stirring for seven hours, after which it was filtered and left to cool to room temperature. After 12 h, brown-yellow crystals of the titls compound began to form and were removed by filtration after two days.

### Refinement

Hydrogen atoms attached to N and water H atoms were located from a difference map and their positional and isotropic displacenent parameters were refined. For the water molecule, there is no possible acceptor for the one of the hydrogen atom (H5A). Other H atoms were placed geometrically and were allowed to ride on the parent atom, with C—H = 0.93 Å and O—H = 0.84 Å and with *U*_iso_(H) set to 1.2–1.5 times *U*_eq_(C, O). The uncertainty of the Flack parameter [-0.02 (8) for 1677 Friedel pairs] did not allow the the absolute configuration to be definitively assigned (*S* for C10).

### Crystal data

**Table:**

C_18_H_16_N_2_O_4_·H_2_O	*F*(000) = 720
*M**_r_* = 342.34	*D*_x_ = 1.334 Mg m^−^^3^
Orthorhombic, *P*2_1_2_1_2_1_	Mo *K*α radiation, λ = 0.71073 Å
Hall symbol: P 2ac 2ab	Cell parameters from 6129 reflections
*a* = 8.4214 (3) Å	θ = 2.7–28.3°
*b* = 10.6787 (4) Å	µ = 0.10 mm^−^^1^
*c* = 18.9554 (8) Å	*T* = 100 K
*V* = 1704.65 (11) Å^3^	Block, brown-yellow
*Z* = 4	0.20 × 0.10 × 0.10 mm

### Data collection

**Table:**

Bruker SMART APEXII CCD area-detector diffractometer	3904 independent reflections
Radiation source: fine-focus sealed tube	3587 reflections with *I* > 2σ(*I*)
graphite	*R*_int_ = 0.035
φ and ω scans	θ_max_ = 27.5°, θ_min_ = 2.2°
Absorption correction: multi-scan (*SADABS*; Sheldrick, 1996)	*h* = −10→10
*T*_min_ = 0.618, *T*_max_ = 0.746	*k* = −13→13
15797 measured reflections	*l* = −24→24

### Refinement

**Table:**

Refinement on *F*^2^	Primary atom site location: structure-invariant direct methods
Least-squares matrix: full	Secondary atom site location: difference Fourier map
*R*[*F*^2^ > 2σ(*F*^2^)] = 0.034	Hydrogen site location: inferred from neighbouring sites
*wR*(*F*^2^) = 0.085	H atoms treated by a mixture of independent and constrained refinement
*S* = 1.03	*w* = 1/[σ^2^(*F*_o_^2^) + (0.0474*P*)^2^ + 0.2857*P*] where *P* = (*F*_o_^2^ + 2*F*_c_^2^)/3
3904 reflections	(Δ/σ)_max_ < 0.001
244 parameters	Δρ_max_ = 0.25 e Å^−^^3^
0 restraints	Δρ_min_ = −0.16 e Å^−^^3^

### Special details

**Table:**

Geometry. All e.s.d.'s (except the e.s.d. in the dihedral angle between two l.s. planes) are estimated using the full covariance matrix. The cell e.s.d.'s are taken into account individually in the estimation of e.s.d.'s in distances, angles and torsion angles; correlations between e.s.d.'s in cell parameters are only used when they are defined by crystal symmetry. An approximate (isotropic) treatment of cell e.s.d.'s is used for estimating e.s.d.'s involving l.s. planes.
Refinement. Refinement of *F*^2^ against ALL reflections. The weighted *R*-factor *wR* and goodness of fit *S* are based on *F*^2^, conventional *R*-factors *R* are based on *F*, with *F* set to zero for negative *F*^2^. The threshold expression of *F*^2^ > σ(*F*^2^) is used only for calculating *R*-factors(gt) *etc*. and is not relevant to the choice of reflections for refinement. *R*-factors based on *F*^2^ are statistically about twice as large as those based on *F*, and *R*- factors based on ALL data will be even larger.

### Fractional atomic coordinates and isotropic or equivalent isotropic displacement parameters (Å^2^)

**Table:**

	*x*	*y*	*z*	*U*_iso_*/*U*_eq_	
O4	0.45139 (13)	0.65534 (9)	0.31898 (6)	0.0204 (2)	
H4	0.3667	0.6867	0.3036	0.031*	
O2	0.19336 (12)	−0.24604 (9)	0.26862 (6)	0.0211 (2)	
O3	0.17010 (12)	0.27877 (9)	0.26869 (6)	0.0235 (2)	
H3	0.0984	0.3300	0.2579	0.035*	
N2	0.29712 (15)	0.06525 (11)	0.31452 (6)	0.0170 (2)	
C16	0.43988 (17)	0.53039 (13)	0.31846 (7)	0.0172 (3)	
O1	0.04987 (13)	−0.07062 (10)	0.26999 (7)	0.0294 (3)	
C18	0.55615 (17)	0.33389 (13)	0.34947 (7)	0.0169 (3)	
H18	0.6416	0.2870	0.3689	0.020*	
C17	0.56704 (18)	0.46154 (13)	0.34693 (7)	0.0176 (3)	
H17	0.6590	0.5031	0.3641	0.021*	
C15	0.30669 (18)	0.47039 (13)	0.29074 (7)	0.0184 (3)	
H15	0.2236	0.5181	0.2701	0.022*	
C13	0.42086 (17)	0.26989 (13)	0.32395 (7)	0.0162 (3)	
C12	0.41503 (17)	0.13809 (13)	0.33109 (7)	0.0161 (3)	
H12	0.5068	0.0987	0.3500	0.019*	
C10	0.29777 (18)	−0.06908 (12)	0.32974 (7)	0.0172 (3)	
H10	0.4045	−0.1051	0.3186	0.021*	
C11	0.17062 (18)	−0.13330 (13)	0.28460 (7)	0.0192 (3)	
C3	0.53472 (19)	−0.05160 (14)	0.46542 (7)	0.0207 (3)	
C14	0.29580 (18)	0.34069 (13)	0.29342 (7)	0.0180 (3)	
C9	0.25679 (18)	−0.09299 (14)	0.40849 (7)	0.0211 (3)	
H9A	0.1471	−0.0642	0.4179	0.025*	
H9B	0.2614	−0.1840	0.4181	0.025*	
N1	0.47368 (18)	0.12210 (13)	0.52719 (7)	0.0263 (3)	
C1	0.33771 (19)	0.07978 (15)	0.49523 (8)	0.0249 (3)	
H1	0.2365	0.1185	0.4992	0.030*	
C8	0.5967 (2)	0.04331 (15)	0.50975 (8)	0.0231 (3)	
C7	0.7571 (2)	0.04678 (16)	0.52806 (8)	0.0273 (4)	
H7	0.7982	0.1111	0.5575	0.033*	
C4	0.6373 (2)	−0.14419 (15)	0.43870 (8)	0.0244 (3)	
H4A	0.5984	−0.2079	0.4083	0.029*	
C6	0.85398 (19)	−0.04657 (16)	0.50188 (8)	0.0283 (4)	
H6	0.9633	−0.0471	0.5143	0.034*	
C5	0.7951 (2)	−0.14071 (17)	0.45741 (8)	0.0279 (3)	
H5	0.8653	−0.2031	0.4399	0.033*	
C2	0.36843 (18)	−0.02646 (14)	0.45657 (8)	0.0213 (3)	
O5	0.41346 (18)	0.32658 (13)	0.62788 (7)	0.0344 (3)	
H2A	0.208 (2)	0.0933 (17)	0.2981 (9)	0.023 (4)*	
H5B	0.410 (3)	0.311 (2)	0.6689 (16)	0.055 (8)*	
H1A	0.476 (3)	0.186 (2)	0.5555 (12)	0.041 (6)*	
H5A	0.387 (4)	0.394 (3)	0.6163 (18)	0.083 (11)*	

### Atomic displacement parameters (Å^2^)

**Table:**

	*U*^11^	*U*^22^	*U*^33^	*U*^12^	*U*^13^	*U*^23^
O4	0.0223 (5)	0.0114 (5)	0.0276 (5)	0.0001 (4)	−0.0034 (5)	−0.0001 (4)
O2	0.0241 (5)	0.0119 (5)	0.0274 (5)	−0.0006 (4)	−0.0056 (4)	−0.0014 (4)
O3	0.0216 (5)	0.0120 (5)	0.0369 (6)	0.0004 (4)	−0.0124 (5)	0.0013 (4)
N2	0.0185 (6)	0.0117 (5)	0.0208 (6)	0.0010 (5)	−0.0045 (5)	0.0001 (4)
C16	0.0220 (7)	0.0119 (6)	0.0176 (6)	−0.0012 (5)	0.0018 (6)	−0.0001 (5)
O1	0.0251 (6)	0.0147 (5)	0.0484 (7)	0.0010 (4)	−0.0173 (5)	−0.0028 (5)
C18	0.0151 (7)	0.0157 (7)	0.0199 (6)	0.0019 (6)	−0.0013 (5)	−0.0006 (5)
C17	0.0184 (7)	0.0149 (7)	0.0196 (6)	−0.0024 (6)	−0.0010 (5)	−0.0024 (5)
C15	0.0207 (7)	0.0141 (7)	0.0204 (6)	0.0023 (6)	−0.0031 (6)	0.0020 (5)
C13	0.0188 (7)	0.0122 (6)	0.0177 (6)	0.0001 (5)	0.0000 (5)	−0.0017 (5)
C12	0.0172 (7)	0.0145 (7)	0.0166 (6)	0.0019 (5)	−0.0003 (5)	−0.0013 (5)
C10	0.0189 (7)	0.0101 (6)	0.0226 (7)	0.0007 (5)	−0.0029 (6)	0.0005 (5)
C11	0.0216 (7)	0.0136 (7)	0.0225 (7)	−0.0024 (6)	−0.0040 (6)	0.0029 (5)
C3	0.0255 (8)	0.0201 (7)	0.0165 (6)	−0.0034 (6)	−0.0016 (6)	0.0037 (6)
C14	0.0193 (7)	0.0152 (7)	0.0194 (6)	0.0000 (6)	−0.0028 (5)	−0.0009 (5)
C9	0.0217 (7)	0.0181 (7)	0.0235 (7)	−0.0021 (6)	−0.0009 (6)	0.0018 (6)
N1	0.0332 (8)	0.0243 (7)	0.0215 (6)	−0.0028 (6)	0.0001 (6)	−0.0055 (5)
C1	0.0275 (8)	0.0253 (8)	0.0218 (7)	−0.0003 (7)	0.0020 (6)	0.0003 (6)
C8	0.0306 (8)	0.0233 (8)	0.0155 (6)	−0.0035 (6)	0.0001 (6)	0.0019 (6)
C7	0.0330 (9)	0.0309 (9)	0.0180 (7)	−0.0082 (7)	−0.0047 (6)	−0.0011 (6)
C4	0.0295 (8)	0.0217 (8)	0.0221 (7)	−0.0003 (7)	−0.0034 (6)	0.0000 (6)
C6	0.0234 (8)	0.0387 (9)	0.0227 (7)	−0.0054 (7)	−0.0050 (6)	0.0013 (7)
C5	0.0285 (9)	0.0294 (8)	0.0257 (8)	0.0024 (7)	−0.0012 (6)	−0.0017 (6)
C2	0.0243 (8)	0.0213 (8)	0.0184 (7)	−0.0048 (6)	0.0002 (6)	0.0031 (6)
O5	0.0503 (8)	0.0244 (7)	0.0286 (7)	−0.0047 (6)	0.0015 (6)	0.0015 (5)

### Geometric parameters (Å, °)

**Table:**

O4—C16	1.3379 (17)	C10—H10	1.0000
O4—H4	0.8400	C3—C4	1.407 (2)
O2—C11	1.2562 (18)	C3—C8	1.416 (2)
O3—C14	1.3333 (17)	C3—C2	1.436 (2)
O3—H3	0.8400	C9—C2	1.490 (2)
N2—C12	1.2999 (18)	C9—H9A	0.9900
N2—C10	1.4631 (17)	C9—H9B	0.9900
N2—H2A	0.87 (2)	N1—C1	1.372 (2)
C16—C15	1.395 (2)	N1—C8	1.375 (2)
C16—C17	1.407 (2)	N1—H1A	0.87 (2)
O1—C11	1.2485 (18)	C1—C2	1.375 (2)
C18—C17	1.367 (2)	C1—H1	0.9500
C18—C13	1.4139 (19)	C8—C7	1.395 (2)
C18—H18	0.9500	C7—C6	1.380 (3)
C17—H17	0.9500	C7—H7	0.9500
C15—C14	1.3890 (19)	C4—C5	1.376 (2)
C15—H15	0.9500	C4—H4A	0.9500
C13—C12	1.4148 (19)	C6—C5	1.403 (2)
C13—C14	1.420 (2)	C6—H6	0.9500
C12—H12	0.9500	C5—H5	0.9500
C10—C11	1.5327 (19)	O5—H5B	0.80 (3)
C10—C9	1.553 (2)	O5—H5A	0.78 (3)
			
C16—O4—H4	109.5	O3—C14—C15	122.29 (13)
C14—O3—H3	109.5	O3—C14—C13	117.91 (12)
C12—N2—C10	122.42 (12)	C15—C14—C13	119.80 (13)
C12—N2—H2A	122.9 (12)	C2—C9—C10	111.67 (12)
C10—N2—H2A	114.4 (12)	C2—C9—H9A	109.3
O4—C16—C15	121.28 (13)	C10—C9—H9A	109.3
O4—C16—C17	117.60 (13)	C2—C9—H9B	109.3
C15—C16—C17	121.11 (12)	C10—C9—H9B	109.3
C17—C18—C13	121.60 (14)	H9A—C9—H9B	107.9
C17—C18—H18	119.2	C1—N1—C8	108.72 (13)
C13—C18—H18	119.2	C1—N1—H1A	123.3 (15)
C18—C17—C16	118.93 (13)	C8—N1—H1A	127.9 (15)
C18—C17—H17	120.5	N1—C1—C2	110.49 (14)
C16—C17—H17	120.5	N1—C1—H1	124.8
C14—C15—C16	119.83 (13)	C2—C1—H1	124.8
C14—C15—H15	120.1	N1—C8—C7	130.80 (15)
C16—C15—H15	120.1	N1—C8—C3	107.65 (14)
C18—C13—C12	118.43 (13)	C7—C8—C3	121.55 (15)
C18—C13—C14	118.68 (12)	C6—C7—C8	117.62 (15)
C12—C13—C14	122.89 (13)	C6—C7—H7	121.2
N2—C12—C13	126.78 (13)	C8—C7—H7	121.2
N2—C12—H12	116.6	C5—C4—C3	118.77 (15)
C13—C12—H12	116.6	C5—C4—H4A	120.6
N2—C10—C11	109.03 (11)	C3—C4—H4A	120.6
N2—C10—C9	110.48 (11)	C7—C6—C5	121.64 (15)
C11—C10—C9	107.92 (11)	C7—C6—H6	119.2
N2—C10—H10	109.8	C5—C6—H6	119.2
C11—C10—H10	109.8	C4—C5—C6	121.05 (16)
C9—C10—H10	109.8	C4—C5—H5	119.5
O1—C11—O2	125.77 (13)	C6—C5—H5	119.5
O1—C11—C10	116.93 (12)	C1—C2—C3	105.99 (14)
O2—C11—C10	117.20 (12)	C1—C2—C9	126.92 (14)
C4—C3—C8	119.35 (15)	C3—C2—C9	126.72 (14)
C4—C3—C2	133.49 (15)	H5B—O5—H5A	117 (3)
C8—C3—C2	107.15 (14)		
			
C13—C18—C17—C16	0.3 (2)	C11—C10—C9—C2	−176.14 (12)
O4—C16—C17—C18	177.64 (13)	C8—N1—C1—C2	−0.01 (18)
C15—C16—C17—C18	−2.3 (2)	C1—N1—C8—C7	179.16 (16)
O4—C16—C15—C14	−177.57 (14)	C1—N1—C8—C3	−0.11 (17)
C17—C16—C15—C14	2.4 (2)	C4—C3—C8—N1	178.89 (13)
C17—C18—C13—C12	−177.31 (14)	C2—C3—C8—N1	0.19 (16)
C17—C18—C13—C14	1.6 (2)	C4—C3—C8—C7	−0.5 (2)
C10—N2—C12—C13	−174.50 (13)	C2—C3—C8—C7	−179.16 (14)
C18—C13—C12—N2	175.73 (14)	N1—C8—C7—C6	−179.74 (15)
C14—C13—C12—N2	−3.2 (2)	C3—C8—C7—C6	−0.6 (2)
C12—N2—C10—C11	−162.62 (12)	C8—C3—C4—C5	0.9 (2)
C12—N2—C10—C9	78.95 (17)	C2—C3—C4—C5	179.23 (16)
N2—C10—C11—O1	−31.31 (18)	C8—C7—C6—C5	1.1 (2)
C9—C10—C11—O1	88.72 (15)	C3—C4—C5—C6	−0.4 (2)
N2—C10—C11—O2	152.17 (13)	C7—C6—C5—C4	−0.6 (3)
C9—C10—C11—O2	−87.79 (16)	N1—C1—C2—C3	0.13 (17)
C16—C15—C14—O3	178.95 (13)	N1—C1—C2—C9	−173.23 (14)
C16—C15—C14—C13	−0.4 (2)	C4—C3—C2—C1	−178.63 (16)
C18—C13—C14—O3	179.05 (12)	C8—C3—C2—C1	−0.20 (16)
C12—C13—C14—O3	−2.1 (2)	C4—C3—C2—C9	−5.2 (3)
C18—C13—C14—C15	−1.5 (2)	C8—C3—C2—C9	173.18 (14)
C12—C13—C14—C15	177.34 (14)	C10—C9—C2—C1	105.32 (17)
N2—C10—C9—C2	−57.03 (16)	C10—C9—C2—C3	−66.71 (19)

### Hydrogen-bond geometry (Å, °)

**Table:**

*D*—H···*A*	*D*—H	H···*A*	*D*···*A*	*D*—H···*A*
O4—H4···O2^i^	0.84	1.76	2.5966 (15)	178.
O3—H3···O1^ii^	0.84	1.72	2.5605 (15)	176.
O3—H3···O2^ii^	0.84	2.64	3.1526 (14)	121.
N2—H2A···O3	0.87 (2)	2.082 (19)	2.6642 (15)	123.9 (16)
O5—H5B···O2^iii^	0.80 (3)	2.19 (3)	2.9438 (17)	157 (2)
N1—H1A···O5	0.87 (2)	2.10 (2)	2.9441 (19)	164 (2)

Symmetry codes: (i) *x*, *y*+1, *z*; (ii) −*x*, *y*+1/2, −*z*+1/2; (iii) −*x*+1/2, −*y*, *z*+1/2.

## References

[bb1] Allen, F. H., Kennard, O., Watson, D. G., Brammer, L., Orpen, A. G. & Taylor, R. (1987). *J. Chem. Soc. Perkin Trans. 2*, pp. S1–19.

[bb2] Bruker (2008). *APEX2* and *SAINT* Bruker AXS Inc., Madison, Wisconsin, USA.

[bb3] Dao, V.-T., Gaspard, C., Mayer, M., Werner, G. H., Nguyen, S. N. & Michelot, R. J. (2000). *Eur. J. Med. Chem.* **35**, 805–813. 10.1016/s0223-5234(00)00165-3 11006482

[bb4] Eltayeb, N. E. & Ahmed, T. A. (2005*a*). *J. Sci. Technol.* **6**, 51–59.

[bb5] Eltayeb, N. E. & Ahmed, T. A. (2005*b*). *Sudan J. Basic Sci.* **7**, 97–108.

[bb6] Emge, T. J., Agrawal, A., Dalessio, J. P., Dukovic, G., Inghrim, J. A., Janjua, K., Macaluso, M., Robertson, L. L., Stiglic, T. J., Volovik, Y. & Georgiadis, M. M. (2000). *Acta Cryst.* C**56**, e469–e471.

[bb8] Grant, G. D., Hunt, A. L., Milne, P. J., Roos, H. M. & Joubert, J. A. (1999). *J. Chem. Crystallogr.* **29**, 435–447.

[bb9] Karthikeyan, M. S., Prasad, D. J., Poojary, B., Bhat, K. S., Holla, B. S. & Kumari, N. S. (2006). *Bioorg. Med. Chem.* **14**, 7482–7489. 10.1016/j.bmc.2006.07.015 16879972

[bb13] Sheldrick, G. M. (1996). *SADABS* University of Göttingen, Germany.

[bb10] Sheldrick, G. M. (2008). *Acta Cryst.* A**64**, 112–122. 10.1107/S0108767307043930 18156677

[bb11] Sriram, D., Yogeeswari, P., Myneedu, N. S. & Saraswat, V. (2006). *Bioorg. Med. Chem. Lett.* **16**, 2127–2129. 10.1016/j.bmcl.2006.01.050 16458506

[bb12] Westrip, S. P. (2010). *J. Appl. Cryst.* **43**, 920–925.

